# Endothelial AIP1 Regulates Vascular Remodeling by Suppressing NADPH Oxidase-2

**DOI:** 10.3389/fphys.2018.00396

**Published:** 2018-04-20

**Authors:** Jiqin Zhang, Chaofei Chen, Li Li, Huanjiao J. Zhou, Fenghe Li, Haifeng Zhang, Luyang Yu, Yuxin Chen, Wang Min

**Affiliations:** ^1^Center for Translational Medicine, The First Affiliated Hospital, Sun Yat-sen University, Guangzhou, China; ^2^Department of Pathology and The Vascular Biology and Therapeutics Program, Yale University School of Medicine, New Haven, CT, United States; ^3^Institute of Genetics, Institute of Genetics and Regenerative Biology, College of Life Sciences, Zhejiang University, Hangzhou, China; ^4^Department of Laboratory Medicine, Nanjing Drum Tower Hospital, Nanjing University Medical School, Nanjing, China

**Keywords:** AIP1, NOX2, reactive oxygen species, vascular remodeling, neointimal hyperplasia

## Abstract

**Objective:** AIP1 expression is downregulated in human atherosclerotic plaques and global deletion of AIP1 in mice exacerbates atherosclerosis in ApoE-KO mouse models. However, the direct role of AIP1 in endothelium, vascular remodeling and associated vascular diseases has not been determined.

**Approach and Results:** We used endothelial cell (EC)-specific AIP1-deficient (AIP1-ECKO) mice to define the role of AIP1 in vascular remodeling and intima-media thickening in a mouse carotid artery ligation model characterized by both neointimal hyperplasia and inward vessel remodeling. Compared to WT littermates, AIP1-ECKO mice had 2.2-fold larger intima area and 4.4-fold thicker intima as measured by intima/media ratio in arteries with more proliferating vascular smooth muscle cells (VSMCs) at week 2–4 post-injury. Increased reactive oxygen species (ROS) in endothelium at early time points induced inflammation and vessel dysfunction in AIP1-ECKO prior to VSMC accumulations. Moreover, knockdown of AIP1 in human EC enhanced ROS generation which was attenuated by co-silencing of NOX2. Mechanistically, AIP1 via its proline-rich region binds to the SH3 domain of cytosolic subunit p47phox to disrupt formation of an active NOX2 complex, attenuating ROS production.

**Conclusion:** Our study supports that AIP1 regulates vascular remodeling with intima-media thickening by suppressing endothelial NOX2-dependent oxidative stress.

**Highlights:**

• In a carotid ligation model, endothelial cell (EC)-specific AIP1-deficient (AIP1-ECKO) mice had much larger media area, thicker vessel wall and augmented neointima formation.
• Increased production of reactive oxygen species in vascular EC at early time points concomitant with vessel dysfunction in AIP1-ECKO.
• AIP1 via its proline-rich region binds to the SH3 domain of cytosolic subunit p47phox to disrupt formation of an active NOX2 complex, attenuating ROS production.

## Introduction

The blood vessel wall is made of three tissue layers: the intima is comprised of a single layer of endothelium, the media is composed of vascular smooth muscle cells (VSMCs) and the extracellular matrix synthesized by VSMCs, and the external consists of collagen fibers, fibroblasts, immune cells, and nerves (Libby, 2002; Majesky, 2007; Bennett et al., 2016). The vasculature can sense changes in hemodynamic stimuli and integrate these signals to transduce intra- and intercellular communication that drive structural and functional changes in a process called vascular remodeling (Korshunov et al., 2007; Mozaffarian et al., 2015). Dynamic vascular remodeling is essentially adaptive under physiological conditions, but also contributes to the pathogenesis of intima-media thickening (IMT) and neointima formation (Korshunov et al., 2007; Mozaffarian et al., 2015). Clinically, neointima formation is a common characteristic feature of various forms of vascular diseases, such as restenosis, atherosclerosis and vein graft disease (Korshunov et al., 2007; Mozaffarian et al., 2015).

Unlike skeletal and cardiac muscle cells that are terminally differentiated, VSMCs keep a high degree of plasticity both *in vivo* and *in vitro*. Upon injury such as after angioplasty, stenting or in vascular diseases, VSMCs dedifferentiate and reduce expression of α-SMA, MYH11, ACTA2 and a series of other conventional ‘VSMC markers’ in normal blood vessels as previously reviewed (Owens et al., 2004). During the so-called ‘phenotypic switching’ process, VSMCs dedifferentiate from a contractile state to a highly proliferative synthetic state. VSMCs undergoing phenotypic switching demonstrate increased rate of migration, proliferation and synthesis of extracellular matrix components and meanwhile they can also acquire macrophage markers and phenotypes (Shankman et al., 2015; Cherepanova et al., 2016). This phenotypic switching plays an important role in a large number of human vascular diseases, including atherosclerosis and restenosis. Although vascular smooth muscle cells (VSMC) that exhibit a high proliferation rate and switch from a contractile to synthetic phenotype that directly contribute to neointima formation, vascular endothelial activation and inflammation are earlier clinical events which are causally linked to VSMC accumulation in several vascular disease models (Ross, 1999; Owens et al., 2004; Pober and Sessa, 2007). Specifically, vascular luminal endothelium benefits blood vessels from preventing the adhesion of leukocytes. Nevertheless, pathological processes and injury activate endothelium, contributing to elevation of adhesion molecules including intercellular adhesion molecule-1 (ICAM-1) and vascular cell adhesion protein 1 (VCAM-1) expression, allowing adhesion and transmigration of leukocytes into the artery and leading to VSMC phenotypic changes and subsequent vascular modeling (Owens et al., 2004; Pober and Sessa, 2007).

Reactive oxygen species (ROS), including hydrogen peroxide, superoxide and peroxynitrite, play a central role in endothelial cell (EC) activation and inflammation. Among them, NADPH oxidases (Lassegue and Griendling, 2010; Konior et al., 2014) and the mitochondrial electron transport chain (Quintero et al., 2006) are the major sources of superoxide in vascular cells. Of note, cross talks between NADPH oxidases and mitochondrial ROS has been described (Lee et al., 2006; Ago et al., 2010; Daiber, 2010). NAD(P)H oxidase (NOX) catalyzes the oxidation of NADPH to NADP^+^ or NADH to NAD^+^ to reduce molecular oxygen to the reactive intermediate superoxide. Of the seven NOX enzyme family members, four have been identified as important sources of ROS in the vasculature: NOX1, NOX2, NOX4, and NOX5. The NOX1-2 and p22phox subunits are membrane bound located in cytoplasmic vesicles and the plasma membrane, associating cytosolic subunits p22phox, p40phox, p47phox, and p67phox upon activation. While NOX5 is Ca^2+^-activated, the activation of NOX1–2 is restrictedly relies on phosphorylation and protein-protein interactions of cytosolic subunits. However, NOX4 is constitutively activated in the absence of cytosolic subunits (Sumimoto, 2008; Konior et al., 2014). Moreover, the expression and activity of NOX enzymes and their regulatory subunits are regulated by stresses and cytokines in blood vessels (Lassegue and Griendling, 2010; Konior et al., 2014). In addition to ROS generated from vascular EC and VSMC, infiltrating macrophages within the arterial wall also express NOX enzymes and represent a source of ROS (Rajagopalan et al., 1996; Castier et al., 2005). Animal model studies have supported that NOX enzymes are required for vascular remodeling. However, the source(s) and regulation of NOX-dependent ROS in vascular remodeling are not fully characterized.

ASK1-interacting protein-1 (AIP1; also called DAB2-interacting protein-1, DAB2IP), is a member of the Ras GTPase-activating protein family identified in our lab (Zhang et al., 2003). In addition to the GAP domain, AIP1 also contains other structural domains including a PH domain and a C2 domain at the N-terminal half while the C-terminal half contains a period-like domain, a proline-rich region and a leucine-zipper (Zhang et al., 2003, 2004, 2007b; Xie et al., 2009, 2010). High expression of AIP1 has been revealed in vascular endothelial cell (EC) and smooth muscle cell (VSMC). Consistent with its multiple structural domains, AIP1 acts as scaffolding protein and mediates several inflammatory signaling pathways induced by TNFα, toll-like receptor-4, VEGFR2 and interferon-gamma. Therefore, AIP1 functions similarly to SOCS (suppressor of cytokine signaling) family proteins as a potent anti-inflammatory protein (AIP) (Wan et al., 2010; Min and Pober, 2011; Yu et al., 2011; Zhang et al., 2015). *In vivo* studies suggest that mice with AIP1 global knockout exhibit enormously enhanced inflammatory responses in angiogenesis, graft arteriosclerosis and atherosclerosis (Zhang et al., 2008; Yu et al., 2011; Huang et al., 2013). Importantly, human genome-wide association studies (GWAS) have identified *DAB2IP*, the gene for AIP1, as a susceptibility gene for early onset of myocardial infarction, abdominal aortic aneurysm, and peripheral vascular disease (Gretarsdottir et al., 2010; Harrison et al., 2012). AIP1 expression is downregulated in human atherosclerotic plaques. Global knockout of AIP1 in mice enhanced atherosclerosis process in an ApoE-deficient mouse model (Huang et al., 2013). However, the direct role of AIP1 in endothelium in vascular remodeling and associated vascular diseases has not been determined.

In the course of studying how AIP1 regulates multiple stress and cytokine signaling pathways, we found that AIP1-deficient artery and aortic EC display much higher ROS production. This unexpected observation prompted us to examine the role of EC-specific AIP1 in NOX regulation and vascular remodeling. Here, we report that AIP1 deletion in vascular EC augments neointimal hyperplasia and inward vessel remodeling in a mouse model. Moreover, AIP1 binds to the cytosolic subunit p47phox and suppresses formation of an active NOX2 complex and ROS production. Our data suggests that AIP1 regulates vascular remodeling by suppressing endothelial NOX-dependent oxidative stress in the vasculature.

## Materials and Methods

### Carotid Artery Ligation

All animal studies were approved by the Yale University Animal Care and Use Committee. AIP1-ECKO mice have been bred to C57BL/6 background for more than 6 generations. Both male and female mice at age of 8–10 weeks were used to select age- and sex-matched WT and AIP1-ECKO mice for experiments. After anesthesia, the left external carotid artery was exposed through a midline cervical incision and ligated with a 6–0 silk suture just proximal to the bifurcation (Rudic et al., 1998, 2000; Tang et al., 2008). For sham operations, a suture was tied around the artery in a non-constricting fashion. The right carotid artery was not ligated and served as an internal control. For morphometry and immunohistochemistry experiments, the left and right common carotid arteries were harvested after perfusion fixation with 10% formalin through the left ventricle and immediately embedded in Optimal Cutting Temperature medium (Tissue-Tek). For medial remodeling studies, the perfusion-fixed vessels were post-fixed overnight in 4% paraformaldehyde and embedded in paraffin for optimal histological details of VSMCs and extracellular matrix. For Western blot, qRT-PCR, and ELISA studies, the right and left common carotid arteries were isolated after saline perfusion and snap frozen.

### Morphometric Analysis

Morphometric analysis was performed from hematoxylin and eosin (H & E) stained sections of Optimal Cutting Temperature (OCT) compound – embedded specimens using computer-assisted microscopy. Internal elastic lamina perimeter, external elastic lamina (EEL) perimeter, and medial thickness at four quadrants were measured and averaged over 10 separate sections using ImageJ software (National Institutes of Health). The number of nuclei in each medial lamellar unit and the number of medial lamellar units continuous around the majority (>50%) of the circumference were counted in H & E-stained sections of paraffin-embedded specimens. The transverse diameter of VSMCs was measured across the nucleus using image analysis software and averaged from 20 cells in sections immunostained for α-SMA (Rudic et al., 1998, 2000; Tang et al., 2008). The medial area staining positive for α-SMA, elastin Van Gieson and Sirius red (as a measure of VSMC contractile protein, extracellular elastin, or extracellular collagen, respectively) was calculated using image analyses software.

For immunostaining, antibodies to BrdU (Abcam) and NOX2 (Cell Signaling Technology), followed by secondary antibody labeled with fluorochrome (Invitrogen). The immunostained slides were observed and photographed by a fluorescence microscope (Zeiss). Cell counting of nuclei surrounded by positive immunostaining was performed under high magnification and averaged from 5 cross-sections for each vessel. The vessel area measurements of the lumen (within the endothelium), intima (between the endothelium and internal elastic lamina, IEL), media (between the IEL and external elastic lamina, EEL), and whole vessel (within the EEL) were calculated from 5 serial crosssections, 150 μm apart for each vessel, using computer-assisted image analysis and NIH Image 1.60^1^ (Tang et al., 2008; Yu et al., 2011).

### Artery Ring Assay

Artery ring assay was performed as we described previously (Zhang et al., 2007a; Dai et al., 2009; Huang et al., 2013). The carotid artery was dissected and cut into cylindrical, 3-mm-long segments. The rings are suspended by two tungsten wires mounted in a vessel myograph system (Danish Myotechnologies, Aarhus, Denmark). The artery was bathed in oxygenated Krebs buffer and submitted to a resting tension of 9.8 mN. After 60 min of equilibration with frequent washings, concentration response curves for phenylephrine (PE) were generated to determine vasoconstrictor responses. To study vasodilator and L-nitro arginine methyl ester (L-NAME) responses, the rings were preconstricted with a submaximal concentration of PE, and L-NAME (100 μM), acetylcholine (ACh) (10^-9^ to 10^-5^ M) or sodium nitroprusside (SNP) (10^-9^ to 3 × 10^-7^ M) was injected at the plateau of the PE-induced contraction.

### Real-Time qPCR

Vessels were cut, immersed in water, centrifuged briefly, and then resuspended in TRIzol reagent (Invitrogen). Total RNA was extracted from tissue sections using RNeasy Kit (QIAGEN) according to the manufacturer’s instructions. Reverse transcription was performed using random hexamer primer and oligo-dT according to the Multiscribe RT system protocol (Applied Biosystems). Real-time RT-PCR reactions were performed using TaqMan 2X PCR Master Mix, TaqMan PCR reagents, and commercially available TaqMan gene expression probes for mouse cytokines, chemokines and adhesion molecules or HPRT. The samples and data were analyzed using iQ5 and its system interface software (Bio-Rad). The expression level of each target gene was normalized to HPRT and the results were given as relative copy numbers.

### Cell Culture, Cytokines, and Transfection

Human and mouse aortic endothelial cells were isolated from cells growing out of tissue explants. Briefly, the thoracic aorta was gently cleaned of peri-adventitial fat and connective tissue and then cut into five small segments (1.0–1.5 mm thick). The aortic segments were placed on Matrigel (BD Biosciences) and incubated in DMEM media supplemented with 15% fetal bovine serum, 50 μg/ml heparin, 30 μg/ml ECGS and penicillin-streptomycin. Once sufficient outgrowth of non-fibroblast-like cells was observed, the tissue fragments were removed. At confluence, the cells were passaged using dispase (Worthington) and then cultured for 2 days in culture medium containing D-valine (Sigma-Aldrich) to eliminate fibroblast cells. The subsequent passages were performed with trypsin-EDTA. Immunohistochemical staining of the endothelial monolayer showed strongly positive expression of the endothelial marker, von Willebrand factor. For all experiments reported in this study, only passages 3–5 of primary cultured cells were used. siRNAs purchased from Santa Cruz or Ambion was resolved to the concentration of 20 μM. siRNA knockdown was performed as described previously (Zhang et al., 2003; Min et al., 2008), modified from the manufacturer’s protocol of Oligofectamine (Invitrogen). For cells in one well of 6-well plate, 2 μl siRNA and 8 μl Oligofectamine were mixed in OPTIMEM I sitting at room temperature for 30 min. HAEC were cultured at 90% confluence in 6-well plates and were transfected with the siRNA-Oligofectamine mixture in OPTIMEM I mentioned above for 12 h followed by adding regular culture medium for the other 36 h. Cells were treated as indicated and harvested. Human and mouse recombinant TNFα (R&D Systems) were all used at 10 ng/ml.

### ROS Detection by Flow Cytometry Analysis, Lucigenin Assay and roGFP Reporter

For measurement of ROS, 5,-6-chloromethyl-2′,7′-dichlorodihydrofluorescein diacetate (CM-H_2_DCFDA, preferentially for H_2_O_2_) and dihydroethidium (DHE, preferentially for superoxide) were used for total intracellular ROS, and dihydrorhodamine 123 (DHR123, preferentially for H_2_O_2_) and MitoTracker Red CM-H_2_XROS (MitoROS, preferentially for superoxide) were used for total mitochondrial ROS. All probes were purchased from Molecular Probes. Cells were loaded with 5 μM of an indicated probe and were incubated at 37°C for 20 min and were immediately detached and subjected to flow cytometry analysis (BD Biosciences). The fluorescence signal was recorded on the FL1 (green) or FL3 (red) channel and analyzed by using BD CellQuest software. As controls, cells were treated with TNF (10 ng/ml), paraquat (1 mM) or H_2_O_2_ (1 mM) for indicated times.

For TNFα treatments, HAECs were transfected with control siRNA, AIP1 siRNA or co-transfected with NOX siRNA. After 48 h transfection, cells were either untreated or treated with TNFα (10 ng/ml for 4 h). ROS fluorescence intensities were quantified by Image J software followed by normalization with green fluorescence.

Lucigenin chemiluminescence assay was used for measure superoxide in tissues and cells. The tissues or cells were washed in phenol red-free DMEM (500 μl/dish). Two aliquots (100 μl/aliquot) were saved for protein determination by Lowry protein assay. Other aliquots of cell suspension were added to wells of the 96-well scintillation plate. Chemiluminescence in cells was initiated by adding 100 μM NADPH and 5 μM lucigenin (Sigma) to each well and was measured at 2 min intervals at room temperature with a plate reader (Bio-Tek). The chemiluminescence counts recorded during a 10 min period were averaged and superoxide production was expressed as counts/mg protein/sec.

For roGFP reporter assay, HAEC were transfected with a ROS reporter expressing a thiol redox-sensitive ratiometric sensor roGFP in the mitochondrial matrix (Mito-roGFP) or in the cytosol (Cyto-roGFP). RoGFP mutant contains two surface-exposed cysteines placed at positions 147 and 204 on adjacent β-strands close to the chromophore. Disulfide formation between the cysteine residues promotes protonation of the chromophore and increases the excitation spectrum peak near 400 nm at the expense of the peak near 490 nm. Due to communications between cytosolic ROS and mitochondrial ROS (Lee et al., 2006; Ago et al., 2010; Daiber, 2010; Nazarewicz et al., 2013b), endogenous ROS can activate both Cyto-roGFP and Mito-roGFP reporter genes. Living cell imaging of reduced (495 nm) and oxidized fluorescence (400 nm) were measured under fluorescent microscopy. Total oxidized and reduced fluorescence intensities were quantified by Image J software followed by normalization by taking the control group as 1.0. The ratio of emission from 400 (oxidized) and 495 (reduced) was calculated to determine the relative redox status of the probe in each cell (Waypa et al., 2010). Arbitrarily, a ROS-positive cell has a ratio of oxidized/reduced fluorescence intensity ≥ 0.5 whereas a ROS-negative cell has a ratio < 0.5.

### Immunoprecipitation and Immunoblotting

For co-immunoprecipitation assay, cells were washed with PBS and harvested in a cell lysis buffer (30 mM Tris, pH 8.0, 10 mM NaCl,5 mM EDTA, 10 g/l polyoxyethylene-8-lauryl ether, 1 mM 0-phenanthroline,1 mM iodoacetamide, 10 mM NaF, 5 mM orthovanadate, and 10 mM sodium pyrophosphate). After being precleared using 20 μl of protein A/G PLUS-agarose (Santa Cruz Biotechnology Inc.), cell lysates were incubated with the first protein-specific antiserum (e.g., anti-p47phox) overnight at 4°C and then were incubated with 20 μl of protein A/G PLUS-agarose for additional 2 h with rotation. All immunoprecipitated samples were washed with lysis buffer three times and subjected to SDS-PAGE, followed by immunoblot with the second antibody (e.g., NOX2 or AIP1). The chemiluminescence was detected using ECL kit (Merck Millipore).

For immunoblot in artery and culture cells, protein was extracted from homogenized tissues or cells in lysis buffer, and boiled in SDS sample buffer for 10 min. Equal amounts of protein per sample were separated by SDS-PAGE and electrotransferred to polyvinylidene fluoride membranes (Bio-Rad Laboratories). Antibodies against AIP1 were described previously (Zhang et al., 2003). Primary antibodies used included AIP1 (Invitrogen), NOX1, NOX2, NOX4, NOX5 (Abcam), p47phox, p22phox (Cell Signaling Technology), FLAG, HA and GAPDH (Sigma).

### Statistical Analyses

All data are expressed as mean ± SEM. Two-tailed, paired *t*-tests and a two-way ANOVA analysis were performed using the Prism software program (GraphPad Software). Differences with *P* < 0.05 were considered to indicate statistical significance.

## Results

### AIP1 Deletion in Vascular EC Enhances Vascular Remodeling and Neointima Formation in Mouse Models

**FIGURE 1:**
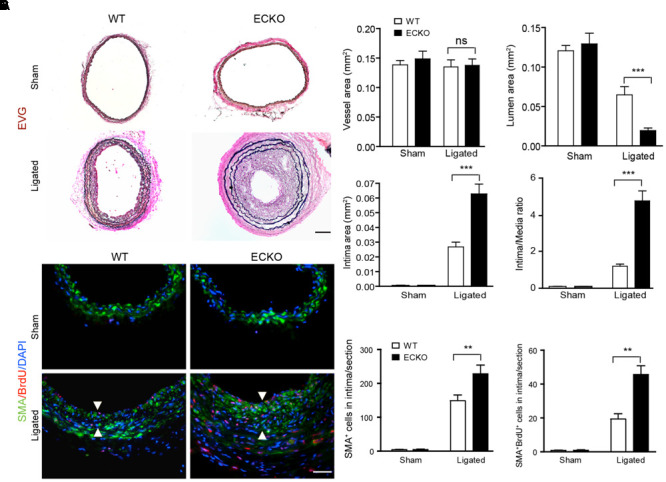
AIP1 deletion in EC enhances ligation-induced vascular remodeling and neointima formation. Carotid arteries from WT or AIP1-ECKO mice were harvested at 3 weeks post-ligation. **(A)** Histological analysis of artery cross sections by Elastica–van Gieson (EVG). Representative photomicrographs are shown. Scale bar: 200 μm. **(B)** Morphometric assessment of whole vessel (within the EEL), artery lumen (within the endothelium), intima (between the endothelium and internal elastic lamina, IEL), media (between the IEL and external elastic lamina, EEL), and intima/media ratios were calculated from 5 serial cross sections. **(C)** AIP1 deletion enhances ligation-induced VSMC accumulation. WT or AIP1-ECKO mice were injected with BrdU at 3 days post-ligation, carotid arteries were harvested at 3 weeks. VSMC proliferation was measured by co-staining with antibodies to α-SMA and BrdU. Representative images are shown in **(C)** with quantifications in **(D)**. Intima are indicated by arrowheads. SMA^+^BrdU^+^ cells were counted as proliferative VSMC. Data are mean ± SEM from 6 mice per group. ^∗∗^*P* < 0.01, ^∗∗∗^*P* < 0.001 comparing WT and AIP1-ECKO groups. ns, no significance. Scale bar: 100 μm **(A)** and 50 μm **(C)**.

To determine the function of AIP1 in vascular remodeling, AIP1-ECKO mice were subjected to a ligation of the left common carotid artery (LCA) in which a mouse LCA was ligated before the carotid bifurcations of external, internal carotid and occipital arteries, and the right common carotid artery (RCA) remained unligated (Supplementary Figure S1A). Consistent with previous findings (Korshunov and Berk, 2003), in response to complete ligation of the vessel near the carotid bifurcation we found that media VSMC rapidly proliferated, followed by extensive neointima formation along an endothelial lining. Thrombosis was not observed except in the most distal part of the vessel adjacent to the ligature. At 3–4 weeks since ligation, luminal narrowing and vascular luminal remodeling could be visualized under microscope at 1–2 mm from the ligation site within ligated LCA, but not unligated RCA (Supplementary Figure S1B). Histological and morphometric analyses indicated that luminal area was reduced by 50% with neointima formation at 3 weeks in WT vessels. Endothelial deletion of AIP1 significantly enhanced neointima formation and luminal narrowing (by 90% at 3 weeks) with no significant alterations in medial and vessel areas (**Figure 1A** with quantifications in **Figure 1B**). Immunostaining indicated that increased accumulations of VSMC (SMA^+^ cells) in AIP1-ECKO were observed after ligation. VSMC proliferation in the neointima was further confirmed by measuring 5-bromo-2′deoxyuridine (BrdU) incorporation in grafts from WT and AIP1-ECKO mice (described in Methods). Proliferative VSMC (SMA^+^ BrdU^+^ cells) were not observed in unligated vessels but were detected in the neointima of ligated WT vessels. Compared to ligated WT, AIP1-ECKO vessels showed an increased number of SMA^+^ BrdU^+^ cells in both neointima and media (**Figure 1C** with quantification in **Figure 1D**). Taken together, these findings indicate that AIP1 deletion augments ligation-induced VSMC proliferation and neointima formation.

### AIP1-ECKO Enhances Ligation-Induced EC Dysfunction Prior to VSMC Accumulations

**FIGURE 2:**
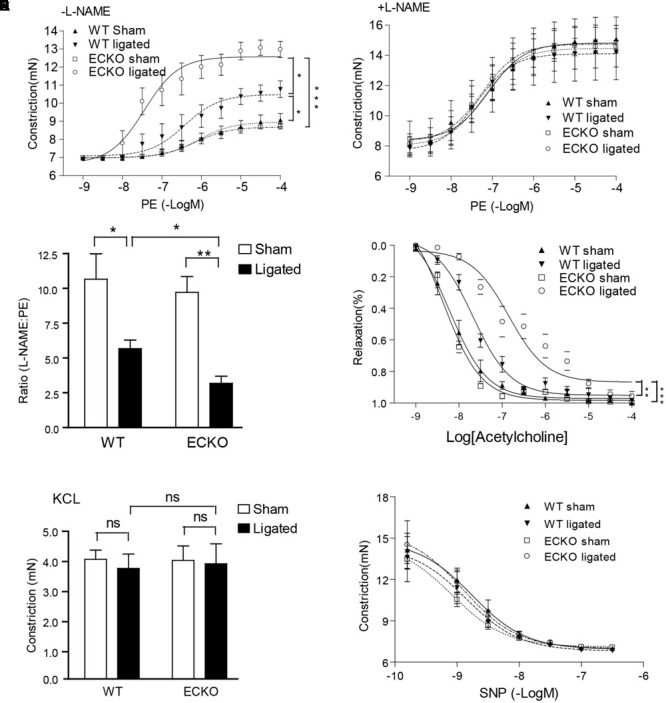
AIP1-ECKO enhances ligation-induced EC dysfunction prior to VSMC accumulations. Three days post-ligation, carotid arteries were harvested for vessel function assays. **(A)** Common carotid artery from AIP1-ECKO mice showed an enhanced response to PE. Common carotid artery rings were contracted with PE at a full range of doses (10^-9^ to 10^-4^ mol/L). Constriction force (mN) is shown. **(B)** AIP1-ECKO had reduced basal NO. Common carotid artery rings were incubated with the NOS inhibitor L-NAME to remove basal NO synthesis and then contracted with PE as described in **(A)**. **(C)** Ratio of EC_50_ to PE in the presence versus absence of L-NAME (100 μmol/L) was shown. **(D)** Arteries from AIP1-ECKO alter the response to Ach. Artery rings were precontracted with PE and then relaxed with Ach at a full range of doses (10^-9^ to 10^-4^ mol/L). % of relaxation is shown. **(E)** AIP1-ECKO had normal vessel constriction in response to KCl. Common carotid artery rings were contracted with 50 mmol/L KCl. **(F)** AIP1-ECKO had no effects on vessel relaxation to the NO donor drug SNP. Common carotid artery rings were incubated with a NOS inhibitor L-NAME to remove basal NO synthesis followed by a pre-contraction with PE as mentioned above and were then relaxed with SNP at a full range of doses (10^-9^ to 10^-6^ mol/L). Data are presented are mean ± SEM, with *n* = 5 animals and eight artery rings per animal. ^∗^*P* < 0.05, ^∗∗^*P* < 0.01, ^∗∗∗^*P* < 0.001.

It has been shown that vascular EC dysfunction is an early event which occurs prior to and is causally linked to VSMC accumulation in several vascular disease models including atherosclerosis and graft arteriosclerosis (Koh et al., 2004; Mitchell, 2004). To determine if AIP1 deletion augmented ligation-induced EC dysfunction, we measured vascular reactivity of isolated artery rings at day 3 after ligation via the administration of the vasoconstrictor phenylephrine (PE) and the NOS inhibitor L-NAME (Zhang et al., 2007a; Dai et al., 2009). Ligation increased vasoconstriction in response to PE. AIP1-ECKO artery showed no difference compared to WT artery in the basal PE response compared to WT artery. However, the PE response in ligated AIP1-ECKO artery was greatly enhanced (**Figure 2A**). This difference was due to a reduced NO bioactivity in ligated AIP1-ECKO artery compared to WT artery, because addition of the NOS inhibitor L-NAME (100 μM) to remove endogenous NO tone resulted in a similar PE response between WT and AIP1-ECKO vessels (**Figures 2B,C**). A ratio of the EC_50_ by phenylephrine in the presence vs. absence of L-NAME was twofold lesser for AIP1-ECKO than WT vessels, indicating that AIP1-ECKO artery had reduced NO activity. Conversely, ECKO vessels exhibited reduced relaxation in response to NO-dependent vasodilator acetylcholine (**Figure 2D**). Unligated or ligated WT and AIP1-ECKO showed similar vasoconstrictive activity toward KCl and relaxation in response to the NO donor SNP (**Figures 2E,F**), suggesting normal VSMC function in these mice. We conclude that AIP1 deletion enhanced ligation-induced EC dysfunction.

### AIP1 Deletion Enhances NOX2 Activity and ROS Generation in Arteries

**FIGURE 3:**
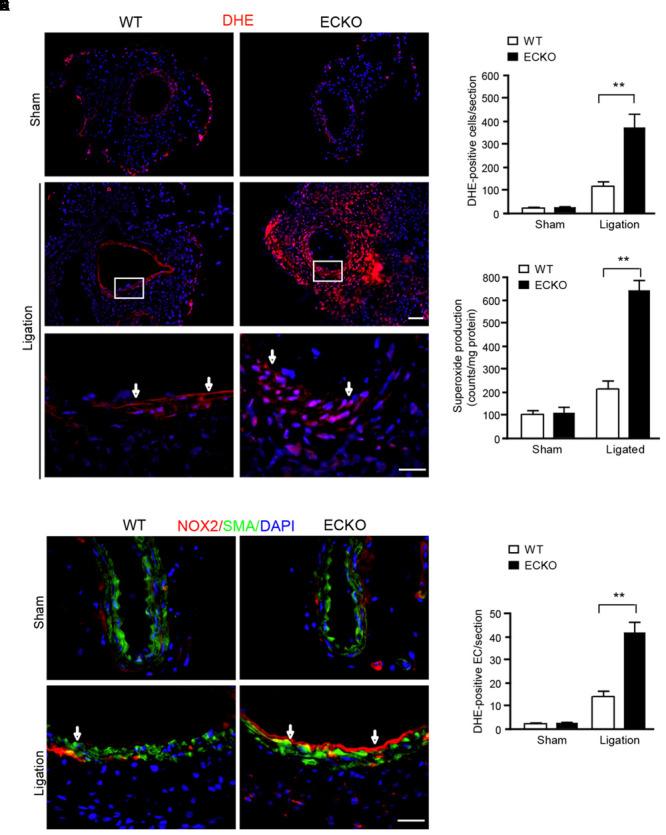
AIP1 deletion enhances NOX2 activity and ROS generation in EC and infiltrated cells. **(A,B)** At day 3 post-ligation, tissue oxidative stress in common carotid arteries was determined by an *in situ* detection of superoxide with dihydroethedium (DHE) fluorescence. Representative images are shown in **(A)** with high power images for the boxed area. DHE positive cells/section is quantified in **(B)**. **(C)** Total NOX activity in carotid artery was determined by lucigenin assay. Data are presented are mean ± SEM, with *n* = 6 animals. ^∗∗^*P* < 0.01. **(D,E)** NOX2 was detected by immunofluorescence staining with anti-NOX2. Smooth muscle actin staining was used to visualize media. Representative images are shown in **(D)** with high power images for the boxed area. NOX2 positive EC/section and DHE positive cells/section are quantified in **(E).** Arrows indicate positive staining in endothelium. Data are presented are mean ± SEM, with *n* = 6 animals and 4 sections per artery. ^∗∗^*P* < 0.01. Scale bar: 50 μm **(A,D)**.

We have recently reported vascular remodeling is associated with ROS (Tang et al., 2008). To determine if AIP1-ECKO mice have augmented ROS during inflammation, we measured ROS production in artery in response to ligation. At day 3 post-ligation, tissue oxidative stress in common carotid arteries was detected by superoxide with dihydroethidium (DHE) fluorescence *in situ*. Ligation increased DHE^+^ staining which was strongly enhanced by AIP1 deletion. DHE^+^ staining appeared throughout the vessel (**Figure 3A** with quantifications in **Figure 3B**). ROS-generating enzyme NADPH oxidases such as NOX1, NOX2, and NOX4 are the major source of ROS in the mouse vasculature. We first measured total NOX activity in arteries by detecting superoxide by lucigenin assay in which NADPH as substrate and lucigenin derived chemiluminescence as detector. Similar to DHE staining, ligation increased NOX activity which was strongly enhanced by AIP1 deletion (**Figure 3C**). We then determined NOX1, NOX2, and NOX4 expression in vessels by Western blotting. NOX1, NOX2, and NOX4 were basally expressed in arteries, and NOX1 and NOX2 were upregulated on day 3 upon ligation. However, only NOX2 was significantly augmented by AIP1 deletion in artery upon ligation (Supplemental Figure S2). Moreover, the induced NOX2 was primarily expressed in endothelium (**Figure 3D** with quantification of NOX2-positive EC in **Figure 3E**). Therefore, NOX2 expression in endothelium was likely upregulated by EC-derived ROS in the AIP1-deficient arteries.

### AIP1 Suppresses NOX2-Mediated ROS Generation in EC

**FIGURE 4:**
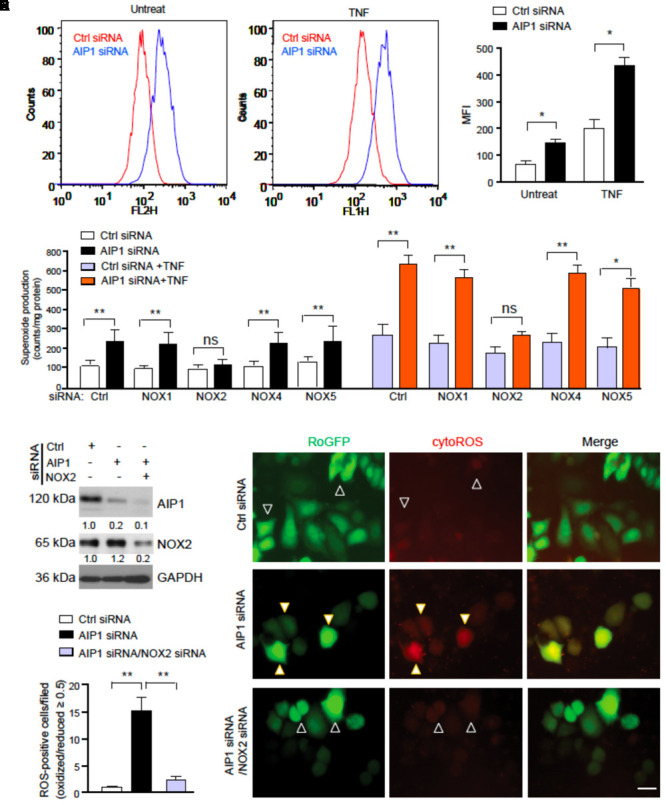
AIP1 regulates ROS generation in EC. **(A,B)** Human aortic EC (HAEC) were transfected with control siRNA and AIP1 siRNA. At 48 h post-transfection, cells were either left untreated or treated with TNFα (10 ng/ml for 4 h) followed by FACS analyses. Intracellular ROS were detected by flow cytometry with specific probe DHE. Representative flow cytometry histograms are shown in **(A)** with quantifications in **(B)**. Data are Mean fluorescence intensity (MFI, mean ± SEM) from four independent experiments are indicated. ^∗^*p* < 0.05. **(C)** HAEC was transfected with control siRNA and AIP1 siRNA in the absence or presence of NOX1, NOX2, NOX4 or NOX5 siRNA. At 48 h post-transfection, cells were either left untreated or treated with TNFα (10 ng/ml for 4 h). Total NOX activity was determined by lucigenin assay. Data are presented are mean ± SEM, with *n* = 3. ^∗∗^*P* < 0.01. **(D–F)** HAEC were transfected with control siRNA or AIP1 siRNA in the absence or presence of NOX2 siRNA. 12 h later, cells were transfected with ROS reporter Cyto-roGFP plasmid. At 24 h post-transfection, living cell imaging of reduced (495 nm) and oxidized fluorescence (400 nm) were measured under fluorescent microscopy. **(D)** Knockdown of AIP1 and NOX2 was verified by Western blotting. Protein bands were quantified by densitometry and fold changes are presented by taking control siRNA as 1.0, *n* = 3. **(E)** Representative images for roGFP are shown. ROS-positive and ROS-negative cells are indicated by open and solid arrowheads, respectively. **(F)** Quantifications of cytoROS-positive cells/field. The ratio of emission from 400 (oxidized) and 495 (reduced) was calculated to determine the relative redox status of the probe in each cell. Arbitrarily, a ROS-positive cell has a ratio of oxidized/reduced fluorescence intensity ≥ 0.5 whereas a ROS-negative cell has a ratio < 0.5. Data are presented as means ± SEM, *n* = 6, ^∗^*P* < 0.05, ^∗∗^*P* < 0.01 using one-way ANOVA with Tukey *post hoc* test. Scale bar: 10 μm **(E)**.

We next determined how AIP1 regulates ROS in EC. To this end, AIP1 was knocked down by siRNAs in human aortic EC (HAEC) and intracellular ROS were detected by flow cytometry with a DHE probe specific for measuring superoxide (Nazarewicz et al., 2013a). AIP1 knockdown in HAEC significantly increased basal ROS. Moreover, AIP1 deletion significantly augmented ROS generation in EC induced by TNFα, a proinflammatory cytokine that was expressed in arteries in the ligation model (**Figure 4A** with quantifications in **Figure 4B** and Supplementary Figure S3 for TNFα expression). To further determine which NOX contributes to ROS generation, NOX1, NOX2, NOX4, or NOX5-mediated superoxide was determined by lucigenin assays in WT and AIP1-deficient HAEC (Of note, human ECs express NOX5). Similar to the DHE staining, AIP1 knockdown augmented both basal and TNFα-induced superoxide production. Moreover, co-repression of NOX2, but not of NOX1, NOX4, or NOX5, diminished AIP1 deletion-augmented basal and TNFα-induced ROS production in human EC (**Figure 4C** and Supplementary Figure S4 for knockdown efficiencies). We further determined NOX2-mediated ROS generation by singe cell live imaging. To this end, control or AIP1-depeleted HAEC were transfected with a ROS reporter expressing a thiol redox-sensitive ratiometric sensor roGFP in the cytosol (Cyto-roGFP) or in the mitochondria (Mito-roGFP), which exhibits different emission fluorescence upon reaction with ROS therefore enabling us to visualize ROS generation in live cells (Waypa et al., 2010). Due to communications between cytosolic ROS and mitochondrial ROS (Lee et al., 2006; Ago et al., 2010; Daiber, 2010; Nazarewicz et al., 2013b), endogenous ROS can activate both Cyto-roGFP and Mito-roGFP reporter genes (Supplementary Figure S5). We imaged individual cells and measured the ratio of emission from 400 (oxidized) and 495 (reduced) to determine the relative redox status of the probe in each cell (Waypa et al., 2010). Results indicated that ROS-positive cells were significantly increased in AIP1-deficient HAEC compared to the control cells. Moreover, NOX2-specific siRNA blocked the ROS production induced by AIP1 depletion (**Figures 4D–F**), suggesting that AIP1 is a potent NOX2 suppressor in EC.

### AIP1 Suppresses Formation of an Active NOX2 Complex and ROS Production

**FIGURE 5:**
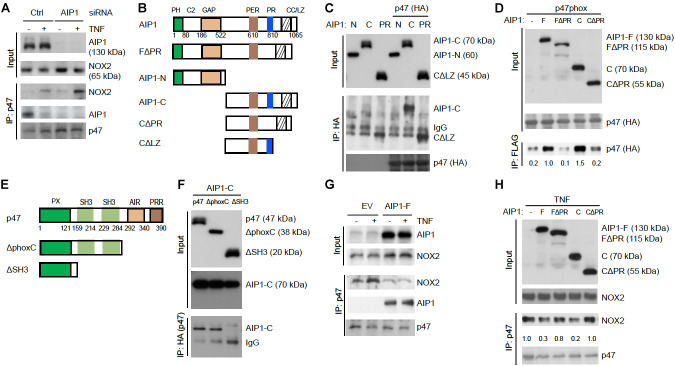
AIP1 blocks NOX2 activity in EC by disrupting the formation of an active NOX2 complex. **(A)** Association of AIP1 with p47phox. HAEC were transfected with control siRNA or AIP1 siRNA. Twenty four hour post-transfection, cells were either left untreated or treated with TNFα (10 ng/ml) for 15 min. AIP1-p47phox and NOX2-p47phox complexes were determined by a co-immunoprecipitation assay followed by Western blot as indicated. **(B)** Schematic diagram for AIP1 structural domains and expression constructs. PH, PH domain; C2, PKC conserved domain; GAP, GTPase-activating protein domain; PER, period-like domain; PRR, proline-rich region; CC/LZ, coiled coil/leucine-zipper domain. Various AIP1 N-terminal truncates (FΔPH and AIP1-N) and C-terminal truncates (AIP1-C; C-PR, C-ΔPR) constructed with a Flag-tagged at the N-terminus are shown. **(C,D)** AIP1 via its PRR binds to p47phox. Various AIP1 truncates were co-transfected with HA-tagged p47phox into HAEC cells as indicated. Associations of AIP1 truncates with p47phox were determined by co-immunoprecipitation with anti-HA (for p47phox) followed by Western blot with anti-Flag (for AIP1 truncates). Immunoprecipitated AIP1 truncates are indicated. **(E)** Schematic diagram for the p47phox structural domains and expression constructs. PX, phosphoinositide-binding structural domain; SH3, Src homology 3 domain that binding to proline-rich region (PRR); AIR, autoinhibitory region; PPR, proline-rich region (PRR). ΔphoxC: a mutant with the deletion of both AIR and PPR; ΔSH3: a mutant with the deletion of the two SH3 domains. **(F)** p47phox via the SH domains bind to AIP1. HA-tagged p47phox truncates were co-transfected with FLAG-tagged AIP1-C into HAEC as indicated. Associations of p47phox truncates with AIP1-C were determined by co-immunoprecipitation with anti-HA (p47phox) followed by Western blot with anti-Flag (AIP1-C). AIP1-C and IgG in the immunoprecipitation are indicated. **(G,H)** AIP1 prevents/disrupts NOX2-p47phox complex formation. HAEC were infected with lentivirus with empty vector (EV), AIP1-F **(E)** and with a truncate **(F)**, and cells were left untreated or treated with TNFα (10 ng/ml for 15 min). Cell lysates were subjected to co-immunoprecipitation assays with anti-p47phox followed by Western blot with anti-NOX2. All experiments were repeated three times.

We next defined the mechanism by which AIP1 regulates NOX2 activity. Formation of the active NOX2 complex by membrane NOX2-p22phox cytosolic subunits and recruited cytosolic p47/p67/p40phox subunits is a key step in NOX2 activation (Quintero et al., 2006; Sumimoto, 2008; Konior et al., 2014). We first examined the NOX2 complex formation in HAEC, in which the NOX2 complex was formed in response to TNFα. Consistent with the ROS levels detected in EC, both basal and TNFα-induced association of NOX2 with p47phox into an active NOX2 complex were increased in AIP1-depleted HAEC. Interestingly, an association of AIP1 with p47phox was also detected in resting WT HAEC that was reduced by TNFα treatment (**Figure 5A**). These data indicate that AIP1 associates with p47phox in resting EC but is dissociated from p47phox in response to stresses. The observation above prompted us to further map the specific domain(s) responsible for the AIP1-p47phox interactions. AIP1 contains a PH, a C2 and a GAP domain in the N-terminal half, and contains a period-like domain, a proline-rich region (PRR), coiled-coil domain (CC), and leucine zipper motif (LZ) in the C-terminal half (**Figure 5B**) (Zhang et al., 2003; Min et al., 2008). A co-immunoprecipitation assay showed that p47phox bound to full-length AIP1 (AIP1-F) and AIP1-C containing the PRR, but not FΔPR or CΔPR (with an internal deletion of the PRR) (**Figures 5C,D**), suggesting that the PRR within the C-terminal half of AIP1 was essential for the interaction of AIP1 with p47phox.

It is known that interactions between the proline-rich region (PRR) of p22phox and the SH3 domain on p47phox are critical for the recruitment of cytosolic subunits (including p47phox) to a membrane p22phox/NOX2 complex (Sumimoto, 2008; Konior et al., 2014). We performed a domain mapping for the role of the p47phox SH3 domain in AIP1-p47phox interactions. AIP1 associated with the p47phox full-length and mutant p47 with a deletion of the phox domain, but not with the mutant p47phox with a deleted the SH3 domain (**Figures 5E,F**). These data indicate that AIP1 via its PRR binds to the SH3 domain of p47phox. We then tested if AIP1 disrupted formation of the p47phox-NOX2 complex formation. Overexpression of a full-length AIP1 (AIP1-F) into HAEC by a lentiviral system blunted basal and TNFα-induced NOX2-p47phox associations (**Figure 5G**). Moreover, expression of AIP1-C containing the PRR, but not FΔPR or CΔPR (with an internal deletion of the PRR), attenuated TNFα-induced formation of the NOX2-p47phox complex (**Figure 5H**). These data suggest that AIP1 via its PRR competes with p22phox for the association with the p47phox subunit.

### The Binding of AIP1 to NOX2 Is Critical for AIP1 in Suppressing ROS Production

**FIGURE 6:**
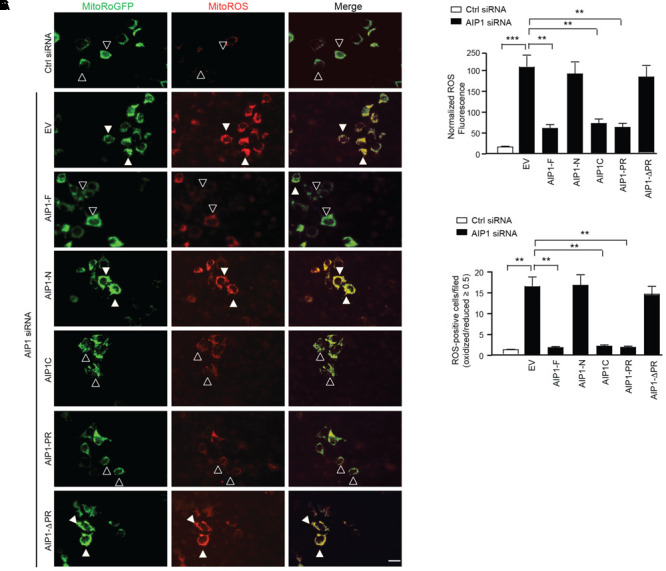
The binding of AIP1 with p47phox is critical for the inhibitory effect of AIP1 on NOX2-mediated ROS generation in EC. **(A)** HAEC were transfected with control siRNA or AIP1 siRNA. 12 h later, cells were co-transfected with various AIP1 truncates and a ROS reporter Mito-roGFP plasmid. 24 h post-transfection, living cell imaging of reduced (495 nm) and oxidized fluorescence (400 nm) were measured under fluorescent microscopy. Representative images are shown. The ratio of emission from 400 (oxidized) and 495 (reduced) was calculated to determine the relative redox status of the probe in each cell. ROS-positive and ROS-negative cells are indicated by solid and open arrowheads, respectively. **(B)** Quantification of normalized ROS fluorescence intensity. Oxidized and reduced fluorescence intensities were quantified by Image J software followed by normalization by taking the control siRNA group as 1.0. **(C)** Quantifications of mitochondrial ROS-positive cells/field. Data are presented as means ± SEM, *n* = 6, ^∗∗^*P* < 0.01, ^∗∗∗^*P* < 0.001 using one-way ANOVA with Tukey *post hoc* test. Scale bar: 10 μm.

Finally, we determined if the binding of AIP1 with p47phox is sufficient to block ROS production. To this end, AIP1 truncates were co-transfected with Mito-roGFP into AIP1-depeleted HAEC and cellular ROS was measured by living cell imaging. Consistent with the binding activity of AIP1 with p47phox, AIP1-F, AIP1-C and AIP1-PR, but not AIP1-N or FΔPR, significantly attenuated ROS production in AIP1-depeleted HAEC (**Figure 6A** with quantifications in **Figures 6B,C**). These data suggest that AIP1 suppresses ROS production in HAEC by preventing formation of the p47phox-NOX2/p22phox complex.

## Discussion

In the present study, we demonstrate that EC-specific deletion of AIP1 (AP1-ECKO) augments vascular remodeling characterized by increased neointimal hyperplasia and enhanced inward vessel remodeling in a mouse carotid artery ligation model of inflammation. AIP1 deficiency enhances ligation-induced EC dysfunction prior to VSMC accumulations; this is correlated with enhanced NOX2 expression and ROS generation in endothelium. We further show that AIP1-deficienct EC increases basal and TNFα-induced ROS production which can be prevented by NOX2-specific siRNA. Moreover, we have uncovered AIP1 as a key negative regulator of NOX2 and defined a novel mechanism for NOX2 regulation. Our study supports that AIP1 suppresses NOX2-dependent oxidative stress in the vasculature to modulate vascular remodeling. These findings may have relevance to design clinical interventions since GWAS has identified AIP1 (the *DAB2IP gene*) as a susceptibility gene for early onset of myocardial infarction, abdominal aortic aneurysm, peripheral vascular disease and pulmonary embolism.

Both cytosolic and mitochondrial ROS play critical roles in flow-induced vascular inflammation and remodeling, although the exact source of ROS had not been determined (Tang et al., 2008). ROS not only alter endothelium-dependent vascular relaxation through interaction with NO, but also oxidize eNOS co-factor BH4, causing a deficiency of BH4 and pathogenic uncoupling of eNOS as well as oxidizing protein to form nitrotyrosine, an indicator of peroxynitrite. We detect enhanced NOX2 expression and ROS production in the AIP1-deficient arteries. Concomitantly, AIP1-deficient artery exhibits reduced NO level with attenuated vascular activity at the earlier phase of vascular remodeling. Furthermore, ROS-initiated inflammation is a necessary precursor for vascular remodeling in the carotid ligation model (Tang et al., 2008). Indeed, we could detect inflammation in the adventitia and neointima for gene expression of inflammatory molecules and infiltration of macrophages which are significantly augmented in AIP1-ECKO vessels in response to ligation. Besides endogenous vascular cells are significant sources of superoxide, infiltrating macrophages within the vascular wall may also represent a source of ROS (Rajagopalan et al., 1996). Nevertheless, our study clearly defines a mechanism for the critical role of EC-derived ROS in promoting inflammation, EC dysfunction and neointima formation.

**FIGURE 7:**
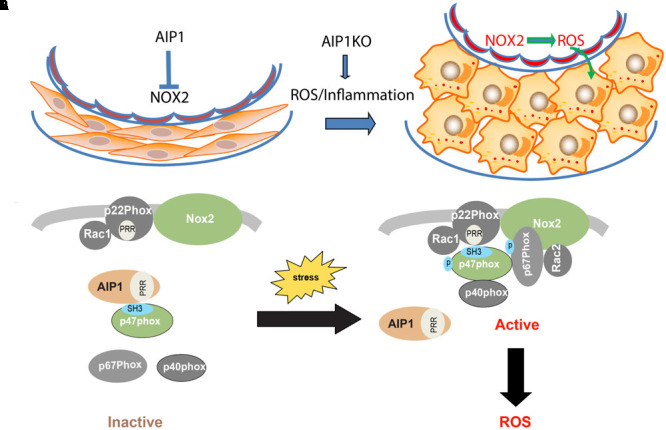
**(A)** Model for AIP1-regulated EC dysfunction and neointima formation. In resting vessel, AIP1 acts as an inhibitor of NOX2 to remain normal endothelial function and VSMC contractile phenotype. Under pathological conditions with increased ROS and inflammation, NOX2 is activated and resulted ROS induce VSMC phenotypic switching from contractile/differentiated state to synthetic/dedifferentiated state, leading to neointima formation. AIP1 deletion further augmented these responses by enhancing NOX2/ROS. **(B)** Mechanism for AIP1-mediated inhibition on NOX2. In resting EC, AIP1 via its PRR associates the SH3 domain of p47phox subunit and prevents recruitment of the cytosolic complex p47phox/p67phox/p40phox to the membrane NOX2/p22phox/Rac1 complex. Stresses such as inflammatory cytokines induce dissociation of AIP1 from p22phox, leading to formation and activation of the NOX2 complex and ROS generation. AIP1 deletion augments these responses and enhances ROS production in EC (see text for details). P, phosphorylation site; PRR, proline-rich region; SH3, SH3 domain.

Increased ROS and inflammation induce endothelial dysfunction, which in turn leads to VSMCs proliferation and neointima formation (Griendling and Alexander, 1997; Libby, 2002; Bennett et al., 2016). Increased inflammation and ROS could also directly modulate ‘phenotypic switching’ process, i.e., dedifferentiation from a contractile state to a synthetic state with increased rate of migration, proliferation and synthesis of extracellular matrix components (Owens et al., 2004). It has been known that modulation of serum responsive factor (SRF) transcription is a mechanism to regulate VSMC genes responsible for contraction (Horita et al., 2016). Inflammatory cytokines such as TNF-α) and IL-1β stimulate the phenotypic switch from the contractile type to the synthetic type by regulating the SRF complex (Bennett et al., 2016). Therefore, the increased ROS and inflammation in AIP1-deficient aorta likely induce VSMC phenotypic switch and proliferation, leading to neointima formation and vascular remodeling (**Figure 7A**: Model for AIP1-regulated EC dysfunction and neointima formation).

The endothelial abundant NOX include NOX1, NOX2, NOX4 and NOX5. Of note, NOX5 is expressed in primates but not in rodents (Sumimoto, 2008). NOX4 is constitutively activated to produce ROS with no presence of phox cytosolic subunits (Nisimoto et al., 2010), while NOX1-2 enzymes are activated in response to stress stimuli and proinflammatory cytokines (Bedard and Krause, 2007). We observe that AIP1-deficient EC have increased both basal and TNFα-induced ROS production. Moreover, knockdown of NOX2, but not of NOX1, NOX4, or NOX5, abolishes the augmented ROS production in AIP1-depleted human EC. These data suggest that NOX2 is a primary target of AIP1 in vascular endothelium. The activity of NOX2 is regulated by their regulatory subunits p22phox, p40phox, p47phox and p67phox and other proteins such as Rac, ClC-3, Poldip2, and protein disulfide isomerase (Sumimoto, 2008; Pendyala et al., 2009). However, a key step in NOX activation is the recruitment of cytosolic subunits (p47phox and p67phox) to cytoplasmic membrane by p22phox through interactions between the proline-rich region (PRR) of p22phox and the SH3 domain on p47phox. An important finding from this study is the mechanism for the regulation of NOX enzymes by AIP1. Specifically, we demonstrate that AIP1 via its proline-rich region (PRR) binds to the SH3 domain of the cytosolic subunit p47phox to disrupt formation of the active NOX2 complex. Given that interactions between the PRR of p22phox and the SH3 domain on p47phox is critical for the recruitment of cytosolic subunits (p47phox and p67phox) to membrane p22phox/NOX2 complex (Sumimoto, 2008; Pendyala et al., 2009), our data support an explanation that AIP1 may compete with p22phox for binding to p47phox. Interestingly, AIP1 is associated with p47phox in resting EC and dissociated from p47phox in response to stresses (e.g., TNFα). These data suggest that AIP1 prevents/or disrupts formation of the NOX2 complex to attenuate NOX2 activity (**Figure 7B**: A model for AIP1-mediated inhibition of the NOX2 complex). It is known that ROS can induce NOX2 expression, forming a positive feedback loop for the NOX2-ROS axis (Lassegue and Griendling, 2010; Konior et al., 2014), consistent with AIP1 deficient EC and artery exhibiting enhanced NOX2 expression and ROS production. Recruitment of cytosolic subunits (p47phox and p67phox) to a membrane p22phox/NOX complex represents a common mechanism for NOX members. Therefore, AIP1 is a novel type of cellular regulator in regulating NOX enzymes. Of note, activation of NOX enzymes is also dependent on the phosphorylation of p47phox at sites located between Ser303 and Ser379 within the AIR and PRR (Raad et al., 2009). We have previously shown that AIP1 could recruit phosphatase PP2A to the C-terminal domain of ASK1 to dephosphorylate of ASK1 at pSer967 (Min et al., 2008). Therefore, it will be interesting to next examine if AIP1 recruits PP2A to the NOX2 complex to disrupt/inactivate NOX2. AIP1 is originally discovered as an ASK1-interacting protein, however, it functions as a scaffolding protein in regulating multiple stress signaling pathways. This role may be attributed to its multiple functional domains responsible for associations with a variety of signaling proteins. Of note, NOX2-derived ROS have been implicated in stress and inflammatory signaling pathways, including TNFα, VEGF and interferon-γ (Yazdanpanah et al., 2009; Lassegue and Griendling, 2010; Kim et al., 2017). Our current study suggests that AIP1 may act as a mediator in the crosstalk between NOX2 and the cytokine signaling pathways, highlighting the important role of AIP1 as a broad anti-inflammatory protein in the vasculature. Furthermore, expression of AIP1 by gene therapy or delivery of peptides derived from the AIP1 PRR should be studied as potential therapeutic approaches for the treatment of vascular diseases.

## Author Contributions

JZ, LL, CC, YC, HJZ, FL, HZ, and LY: designed and performed the experiments. WM: designed and performed the experiments and wrote the manuscript.

## Conflict of Interest Statement

The authors declare that the research was conducted in the absence of any commercial or financial relationships that could be construed as a potential conflict of interest.

## Footnotes

1
http://rsbweb.nih.gov/nih-image/
