# Correction: Endothelial AIP1 regulates vascular remodeling by suppressing NADPH Oxidase-2

**DOI:** 10.3389/fphys.2025.1641354

**Published:** 2025-08-04

**Authors:** Jiqin Zhang, Chaofei Chen, Li Li, Huanjiao J. Zhou, Fenghe Li, Haifeng Zhang, Luyang Yu, Yuxin Chen, Wang Min

**Affiliations:** ^1^ Center for Translational Medicine, The First Affiliated Hospital, Sun Yat-sen University, Guangzhou, China; ^2^ Department of Pathology and The Vascular Biology and Therapeutics Program, Yale University School of Medicine, New Haven, CT, United States; ^3^ Institute of Genetics, Institute of Genetics and Regenerative Biology, College of Life Sciences, Zhejiang University, Hangzhou, China; ^4^ Department of Laboratory Medicine, Nanjing Drum Tower Hospital, Nanjing University Medical School, Nanjing, China

**Keywords:** AIP1, NOX2, reactive oxygen species, vascular remodeling, neointimal hyperplasia

There was a mistake in Figure 5H as published. The NOX2 blot in the IP:p47 bracket was misplaced. A revised Figure 5 with the corrected Figure 5H appears below.

**FIGURE 5:**
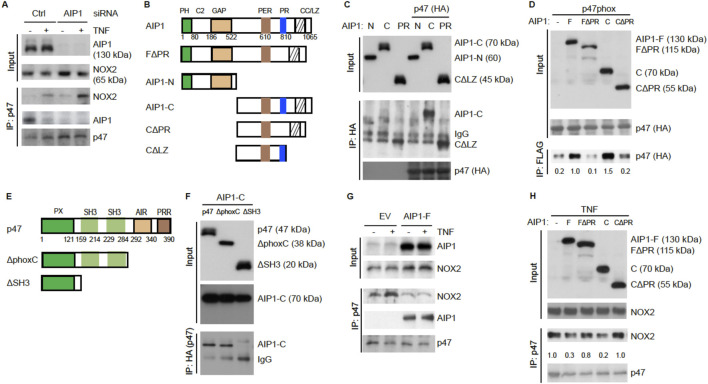
AIP1 blocks NOX2 activity in EC by disrupting the formation of an active NOX2 complex. **(A)** Association of AIP1 with p47phox. HAEC were transfected with control siRNA or AIP1 siRNA. Twenty four hour post-transfection, cells were either left untreated or treated with TNFα (10 ng/ml) for 15 min. AIP1-p47phox and NOX2-p47phox complexes were determined by a co-immunoprecipitation assay followed by Western blot as indicated. **(B)** Schematic diagram for AIP1 structural domains and expression constructs. PH, PH domain; C2, PKC conserved domain; GAP, GTPase-activating protein domain; PER, period-like domain; PRR, proline-rich region; CC/LZ, coiled coil/leucine-zipper domain. Various AIP1 N-terminal truncates (F1PH and AIP1-N) and C-terminal truncates (AIP1-C; C-PR, C-1PR) constructed with a Flag-tagged at the N-terminus are shown. **(C,D)** AIP1 via its PRR binds to p47phox. Various AIP1 truncates were co-transfected with HA-tagged p47phox into HAEC cells as indicated. Associations of AIP1 truncates with p47phox were determined by co-immunoprecipitation with anti-HA (for p47phox) followed by Western blot with anti-Flag (for AIP1 truncates). Immunoprecipitated AIP1 truncates are indicated. **(E)** Schematic diagram for the p47phox structural domains and expression constructs. PX, phosphoinositide-binding structural domain; SH3, Src homology 3 domain that binding to proline-rich region (PRR); AIR, autoinhibitory region; PPR, proline-rich region (PRR). 1phoxC: a mutant with the deletion of both AIR and PPR; 1SH3: a mutant with the deletion of the two SH3 domains. **(F)** p47phox via the SH domains bind to AIP1. HA-tagged p47phox truncates were co-transfected with FLAG-tagged AIP1-C into HAEC as indicated. Associations of p47phox truncates with AIP1-C were determined by co-immunoprecipitation with anti-HA (p47phox) followed by Western blot with anti-Flag (AIP1-C). AIP1-C and IgG in the immunoprecipitation are indicated. **(G,H)** AIP1 prevents/disrupts NOX2-p47phox complex formation. HAEC were infected with lentivirus with empty vector (EV), AIP1-F **(E)** and with a truncate **(F)**, and cells were left untreated or treated with TNFα (10 ng/ml for 15 min). Cell lysates were subjected to co-immunoprecipitation assays with anti-p47phox followed by Western blot with anti-NOX2. All experiments were repeated three times.

The original article has been updated.

