# Supplementary material for: Endothelial AIP1 Regulates Vascular Remodeling by Suppressing NADPH Oxidase-2
Source: Front Physiol. 2018 Apr 20;9:396. doi: 10.3389/fphys.2018.00396 (PMC5921534; doi:10.3389/fphys.2018.00396)
Supplement: Supplementary file 1 [file Data_Sheet_1.pdf]

SUPPLEMENTAL DATA

Endothelial AIP1 regulates vascular remodeling by suppressing NADPH oxidase-2

Jiqin Zhang, Chaofei Chen et al

SUPPLEMENTAL FIGURES

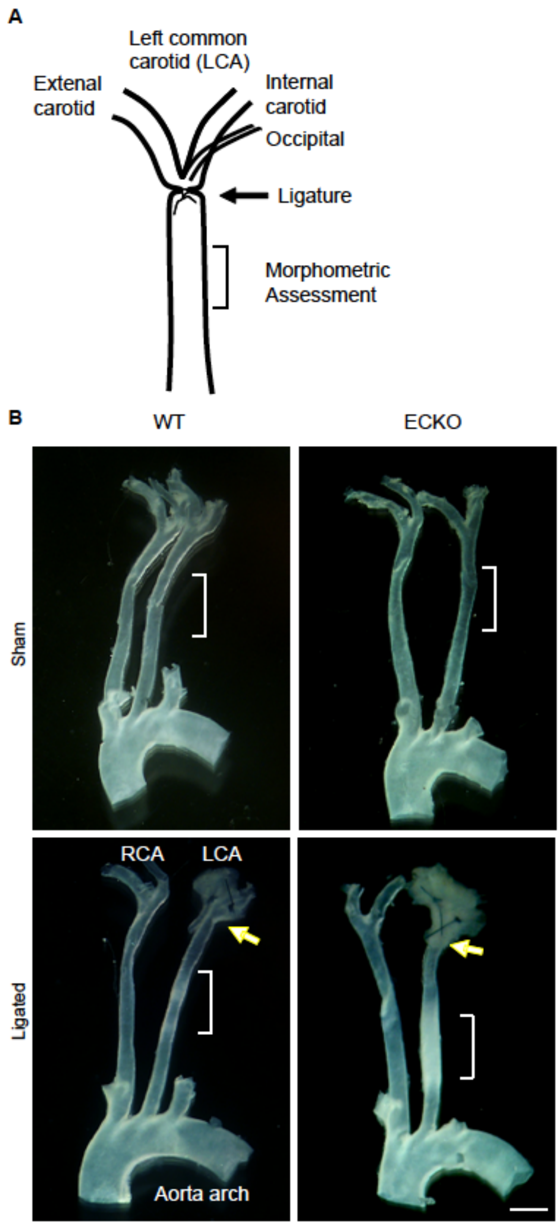

Supplementary Figure 1. **A.** Scheme for a complete ligation of the left common carotid artery (LCA). A mouse LCA was completely ligated before the carotid bifurcation of external, internal carotid and occipital arteries. The right LCA remains unligated. **B.** Carotid arteries from WT or AIP1-ECKO mice were harvested at 3-4 weeks post-ligation. Representative photomicrographs of whole common carotid arteries with aorta arch are shown. The ligation sites are indicated by arrows. Segments with narrowing lumen in the ligated arteries and corresponding sham segments indicated by brackets at 1-2 mm from the ligation site within ligated were used for morphometric assessment. Scale bar: 1 mm.

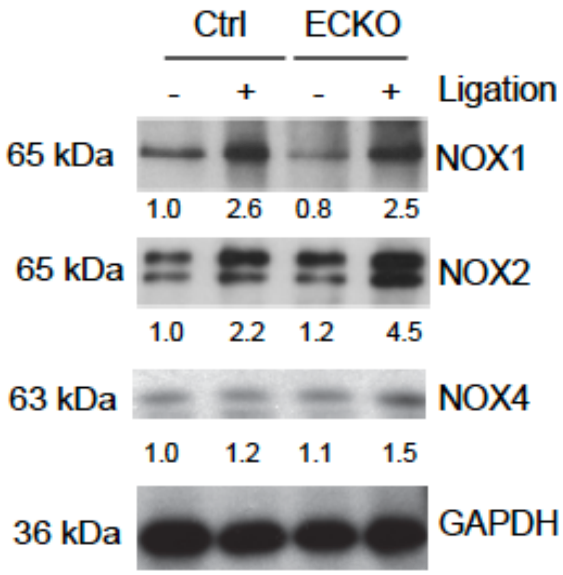

Supplementary Figure 2 AIP1 deletion enhances NOX2 activity. At day 3 post-ligation, carotid arteries were collected and subjected to Western blotting with antibodies as indicated. Protein bands were quantified by densitometry and fold changes are presented by taking WT unligated as 1.0. Data are presented are mean ± SEM, with n =3 animals.

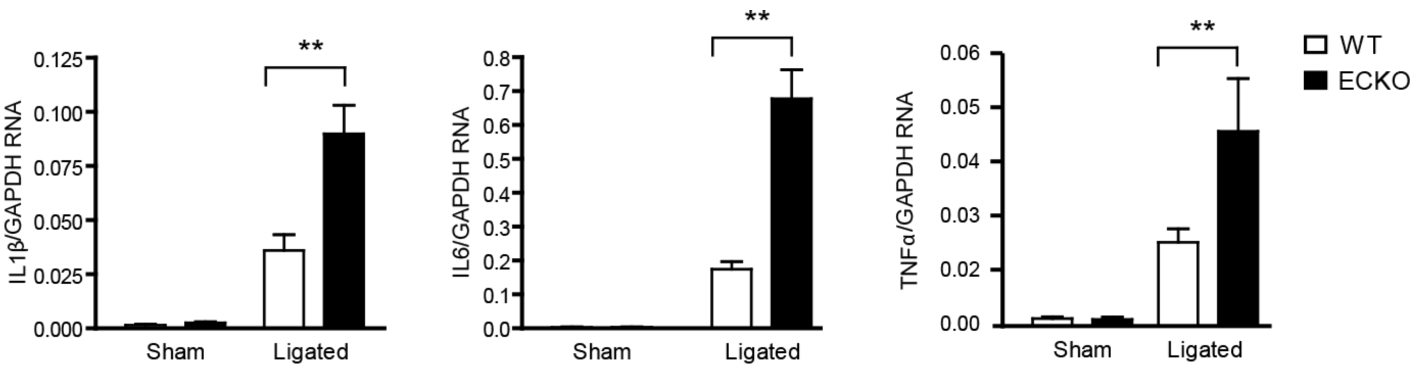

Supplementary Figure 3. **AIP1 deletion enhances gene expression of inflammatory molecules.** Carotid arteries from WT or AIP1-ECKO mice harvested at 3 days post-ligation. Transcripts for cytokines **(**IL-1β, TNF-α and IL-6) from common carotid artery lysates of WT and AIP1-ECKO mice were quantified by qRT-PCR and normalized to GAPDH. Data are mean ± SEM (n=6), $^{**}P<0.01$.

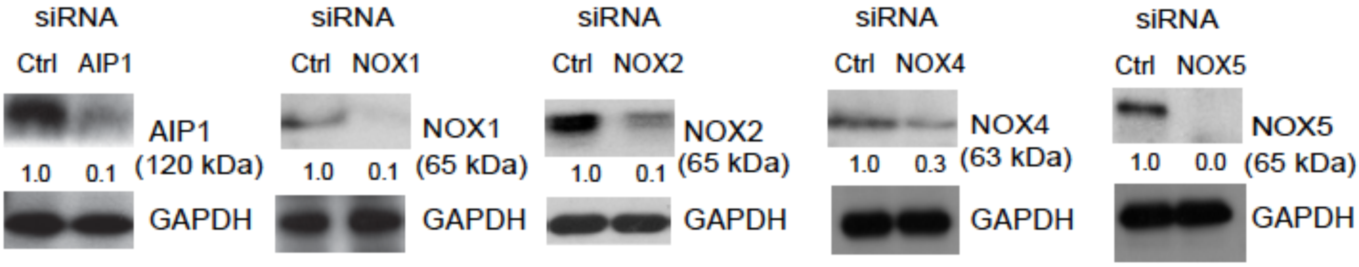

Supplementary Figure 4. Knockdown of AIP1 and NOXs in HAEC. Human aortic ECs (HAECs) were transfected with control siRNA, AIP1 siRNA, NOX1 siRNA, NOX2 siRNA, NOX4 siRNA or NOX5 siRNA. 48 h post-transfection, cells were harvested for detection of protein expression of AIP1, NOX1, NOX2, NOX4 and NOX5 by Western blotting. Protein bands were quantified by densitometry and fold changes are presented by taking control siRNA as 1.0. Data are presented are mean ± SEM, with n =3.

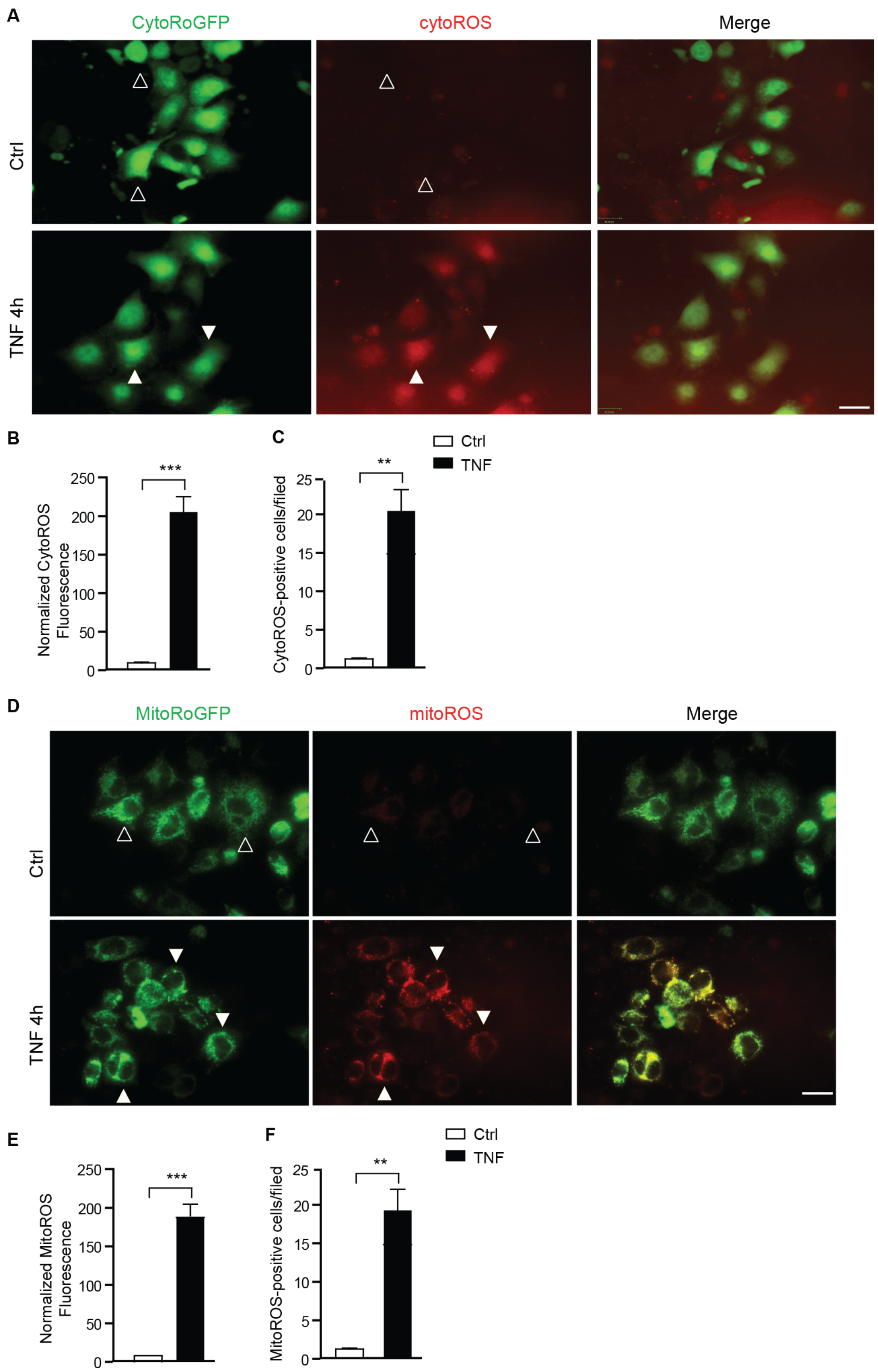

Supplementary Figure 5. Endogenous ROS can activate both Cyto-roGFP and Matrix-roGFP reporter genes. HAEC were transfected with a ROS reporter gene Cyto-roGFP (A-C) or Matrix-roGFP (D-F). 48 h post-transfection, cells were treated with TNF (10 ng/ml for 4 h) prior to living cell imaging of reduced (495 nm) and oxidized fluorescence (400 nm) under fluorescent microscopy. The ratio of emission from 400 (oxidized) and 495 (reduced) was measured to determine the relative redox status of the probe in each cell.

Representative images are shown in A and C. ROS-positive (oxidized/reduced fluorescence intensity ≥0.5) and negative cells (oxidized/reduced fluorescence intensity <0.5) are indicated by solid and open arrowheads, respectively. (B,E) Quantification of normalized ROS fluorescence intensity. Oxidized and reduced fluorescence intensities were quantified by Image J software followed by normalization by taking the control siRNA group as 1.0. (C,F). Quantifications of ROS-positive cells/field. Data are presented as means ± SEM, n=6, **$P<0.01$, *** $P<0.001$ using one-way ANOVA with Tukey post-hoc test. Scale bar: 10 μm.
